# 异基因造血干细胞移植中抗HLA抗体检测质量控制、结果分析和报告中国专家共识（2025年版）

**DOI:** 10.3760/cma.j.cn121090-20250725-00349

**Published:** 2025-10

**Authors:** 中华医学会血液学分会实验诊断学组

## Abstract

抗人类白细胞抗原（HLA）抗体在异基因造血干细胞移植（allo-HSCT）中与移植物植入、造血重建、移植物抗宿主病，制定治疗方案和预后判断均密切相关。抗HLA抗体检测和结果分析不仅受检测技术的局限性和灵敏度影响，还依赖于关键实验步骤的性能确认、全流程质量控制及标准化结果分析步骤等环节的准确性，对检测结果的报告和解读提出了更高要求。在此基础上，中华医学会血液学分会实验诊断学组组织相关专家制定了本共识，旨在使抗HLA抗体检测项目在allo-HSCT临床诊断和治疗中发挥更重要的作用。

## Abstract (en)

Antibodies against human leukocyte antigens（HLA）are closely related to engraftment, hematopoietic reconstitution, graft-versus-host disease, and are crucial for guiding treatment strategies and prognostic assessment in allogeneic hematopoietic stem cell transplantation（allo-HSCT）. The anti-HLA antibody detection and result analysis are not only affected by the inherent limitations and sensitivity of the testing methods, but also depend on the performance validation of key procedures, comprehensive quality, and standardized data analysis, which demand higher standards for result interpretation and reporting. To address these issues, the Experimental Diagnostics Group, Chinese Society of Hematology, Chinese Medical Association has developed this expert consensus, aiming to make the anti-HLA antibody detection play a more important role in the clinical diagnosis and treatment of allo-HSCT.

人类白细胞抗原（human leukocyte antigens, HLA）作为同种异体抗原可产生抗HLA抗体，抗HLA抗体指人群中的个体针对具有人群多态性HLA抗原产生的抗体。抗HLA抗体分为经典的HLA-Ⅰ类抗体（包括HLA-A、HLA-B、HLA-C位点的抗体）和经典的HLA-Ⅱ类抗体（包括HLA-DR、HLA-DQ、HLA-DP位点的抗体）。异基因造血干细胞移植（allogeneic hematopoietic stem cell transplantation, allo-HSCT）前患者血清中检测到抗HLA抗体，称为预存抗HLA抗体（preformed anti-HLA antibodies, pre-Abs）；allo-HSCT后患者检测到移植前阴性或未检测到的HLA抗体类型，称为新生抗HLA抗体（de novo anti-HLA antibodies, dn-Abs）。对于拟接受或已接受单倍体供者（haploidentical donors）、外周血或脐血来源无关错配供者（mismatched unrelated donors）allo-HSCT的患者，其血清中检测到抗HLA抗体，通过潜在供者的HLA分型结果，可以分析是否为供者特异性抗HLA抗体（donor specific anti-HLA antibodies, DSA）[1]。

现国内外研究表明，一定比例拟进行allo-HSCT的患者可检出pre-Abs。一项国内多中心研究显示，在1 663例亲缘单倍体allo-HSCT患者中，pre-Abs的检出率为21.0％[2]。国内另一项研究显示，在1 008例allo-HSCT前血液病患者中，pre-Abs的检出率为24.08％[3]。美国的一项研究显示，在592例接受无关错配allo-HSCT的患者中，pre-Abs的检出率为19.6％[4]。日本的一项多中心研究显示，在343例接受脐血供者allo-HSCT的患者中，pre-Abs检出率为7.29％[5]。国内一项单中心研究报道，allo-HSCT前检测到pre-Abs与移植后发生移植物抗宿主病（graft versus host disease，GVHD）、总生存率密切相关[6]。国外多中心数据显示，allo-HSCT前预存DSA阳性组植入失败、造血重建延缓发生率较DSA阴性组显著升高，生存率显著降低[7]。在对单倍体allo-HSCT前pre-Abs阴性患者的动态随访中发现，75.9％的患者在移植后1个月可检测到dn-Abs，其中28.4％的患者可检测到DSA，持续dn-Abs阳性患者Ⅱ～Ⅳ级急性GVHD（aGVHD）的发生率显著升高，生存率显著降低[8]。70％～85％的免疫性血小板输注无效由HLA-Ⅰ类抗体引起[9]。

抗HLA抗体的检出率取决于实验室技术水平、平均荧光强度（mean fluorescence intensity, MFI）值的阈值[10]，因此，如何制定抗HLA抗体阳性阈值，抗HLA抗体检测结果的分析、判断、报告及解读，以及患者是否需要进行抗HLA抗体治疗、方案的选择和疗效判断等关键临床问题的讨论迫在眉睫。本共识在我国已发表的关于抗HLA抗体检测技术和质量控制等文章的基础上[11]–[13]，对影响抗HLA抗体检测结果关键步骤的质量控制、结果分析、报告解读、动态随访监测时间点等形成共识和推荐建议。

一、抗HLA抗体检测的质量控制

（一）常用抗HLA抗体检测方法

抗HLA抗体检测的常用方法包括细胞学交叉反应法（cross match，XM）和纯化抗原检测法（Luminex技术）。美国组织相容性和免疫遗传学委员会（American Society for Histocompatibility and Immunogenetics，ASHI）2024版标准规定，实验室应具备灵敏度高于补体依赖性细胞毒试验的抗HLA抗体检测方法[14]。Luminex抗体检测方法具有不需要活细胞、操作简便、灵敏度高、可重复性好等优点，近年来在临床上得到广泛应用[15]–[16]。根据纯化抗原的来源分为HLA抗体混合抗原检测试剂（mixed antibodies screen beads, MIX）、群体反应抗体（panel reactive antibodies, PRA）检测试剂、HLA特异性抗原检测试剂（single antigen beads, SAB）。

**专家组推荐**：对于初次接受抗HLA抗体检测的患者，建议采用MIX进行HLA-Ⅰ、HLA-Ⅱ类和MICA抗体的初筛，对初筛结果中可疑阳性或阳性结果进行确定，应采用SAB进行HLA-A、B、C、DR、DQ、DP各位点抗体的抗原类型、MFI值分析。

（二）抗HLA抗体检测项目的性能验证

实验室应按照试剂说明书选择样本类型，如血清或血浆以及标准品、质控品、室间质评样本等。符合率判断标准为定性检测试剂的结果阴、阳性符合率100％（实验室在收集样本时要注意年龄、性别、致敏史的筛选）；在实验室规定时间段内SAB上报阳性抗体类型100％覆盖。批间、批内的重复性要求阴阳性一致，且批内、批间变异系数（CV）<15％[17]。实验室应长期保存性能验证报告中所有内容，如验证方案、检测过程、原始结果、评估报告、记录表单等文件和相关记录。

**专家组推荐**：应优先选择由国家药品监督管理局（National Medical Products Administration，NMPA）批准的注册试剂、仪器及配套的数据分析软件，按照说明书中的性能参数进行性能验证。

（三）涉及抗HLA抗体检测关键实验操作步骤的性能确认

实验室对于使用超出试剂说明书验证范围的操作步骤应进行性能确认，如样本前处理、设立结果判断阈值、改变试剂使用量、改变孵育方法和时间、改变样本保存时间或样本再利用等。实验室应长期保存性能确认报告中的所有内容及必要的引用依据和证明文件。

1. 样本前处理：Luminex法检测结果会受到血清中变性HLA特异性抗体的影响。在检测非常高水平的抗体时，由于存在表位共享或检测微珠饱和、IgM干扰、补体抑制等情况，导致MFI值偏低，不能代表抗体的真实滴度。另外，部分药物治疗，如高剂量丙种球蛋白治疗等，会造成检测结果阴性背景值过高，掩盖真实抗体的MFI值。研究报道，对肾移植患者血清样本采用样本稀释、热孵育、乙二胺四乙酸处理、二硫苏糖醇处理、空微珠吸附、超速离心法等预处理[18]–[20]，有利于减少上述因素对检测结果的影响。预处理方法在allo-HSCT患者应用前应进行性能确认。

2. 抗HLA抗体检测阈值：实验室自行设立的对临床检测结果进行分层的MFI值阈值应进行性能确认。首先应验证在分析软件中预设的阈值与实验室检测结果的一致性，可选择未经输血、年轻男性、体检指标正常的志愿者血清检测抗HLA抗体，以抗HLA抗体MFI值的2～3倍作为阳性阈值，也可利用ASHI组织的抗HLA抗体国际室间能力验证（proficiency testing, PT）项目的反馈结果制定阈值。

苏州大学附属第一医院采用ASHI的PT结果制定实验室判断抗HLA混合抗体初筛结果的阈值表，详见文献[21]中的“实验室HLA混合试剂各微珠MFI及标准化背景（NBG）比值的判断界限值”表格，表格提示，各HLA位点微珠的MFI值及NBG比值均不相同。同时制定SAB结果判断抗HLA抗体饱和值、阳性决定值、阳性阈值、可疑阳性参考值，详见文献[22]中“实验室特异性HLA抗体中位荧光强度（MFI）值判定界限值范围表”。对于设立的MFI值阈值，实验室应制定更新时间及结果评估的标准。

（四）抗HLA抗体检测的质量控制

**专家组推荐：**

①检测前应对申请单进行制定和评估，对样本采集、运输、处理、保存等进行质量控制。

②检测中应对试剂和关键耗材、仪器进行质量控制。应建立室内外质量控制体系，包括质控物的类型、阳性和阴性质控物检测频次、质控记录、在控和失控判断规则、室间质评项目等。

③检测后应对结果分析、样本保存、分析软件和数据库的更新、结果报告、临床解读、报告单发放等进行质量控制。

1. 室内质量控制：

（1）阳性质控物（positive control, PC）及评估：PC应采用阳性质控血清、临床样本、标准品、细胞株等，且应规定在一个时间段内选择的PC至少覆盖试剂包被抗体类型；阴性质控物为商品化标准阴性质控血清。当无商品化PC时，实验室可自行制备质控物[23]。建议选取近期大于5个已知结果临床样本，阳性血清应尽可能多地覆盖各种抗体类型，且大多数MFI值>10 000，与阴性血清样本按照比例混合，使制备的PC的检测结果MFI值处于2 000～4 000，过高的MFI值对临床结果的判断影响小；过低的MFI值重复性差。制备好的PC应进行批内、批间、人员间重复性验证，批间及批内检测结果MFI值CV<15％[17]，则评估通过。应评估制备PC的保存条件、存储温度、储存有效期，保留使用记录。

（2）质控结果分析及判断规则：建议采用累计阳性、阴性质控样本历次实验结果各微珠或抗体类型的平均MFI值*x*、*x*±1*SD*、*x*±2*SD*、*x*±3*SD*制作Levery-Jenning质控图。阴性质控物结果各微珠MFI值处于实验室判断阴性结果的范围，PC与原结果一致，且各微珠或抗体MFI值介于*x*±2*SD*之间，判断为在控。当个别微珠或检测到的抗体的MFI值超出*x*±2*SD*但未超出*x*±3*SD*时，提示警告。当检测人员发现质控物与预期结果不一致或当>50％微珠或检测到的抗体MFI值超出*x*±3*SD*时，需从样本、环境温度、试剂、仪器等方面查找原因，并对整批结果进行核查，确定是否影响结果判读，找到原因后重新进行检测。

2. 室间质量评价：

**专家组推荐：**

①实验室应针对抗HLA抗体检测项目的临床预期用途制定能力验证计划，包括参加机构、频次、样本数量、判断标准等。

②应通过定期参加室间质量评价、PT、实验室间比对等方法实施室间质控。

目前ASHI提供抗HLA抗体检测的PT项目。ASHI 2024版PT评价标准：每年2批次，共10个样本；检测项目涵盖MIX、PRA、SAB；当所有参比实验室上报阳性结果一致率达到90％时，对阳性结果进行评价，当各实验室上报阴性结果一致率达到90％时，对阴性结果进行评价；SAB结果中每个样本的HLA-Ⅰ类和Ⅱ类抗体均允许“多报”≤3个；每个样本的HLA-Ⅰ类和Ⅱ类抗体均允许“漏报”≤2个。

当实验室选择室间比对方法时，首选获得中国合格评定国家认可委员会（China National Accreditation Service for Conformity Assessment，CNAS）认可和相关国际认证实验室或使用相同检测方法的同级别或高级别实验室，确定比对方式的可接受性，规定比对样本数量、频次、评价标准等，并形成比对报告。当实验室选择室内比对方法时，实验室内部至少由两人或两台仪器或两种方法进行盲样检测。

二、抗HLA抗体检测结果分析

**专家组推荐：**

①检测结果导入分析软件的原始数据被有效识别，分析参数至少达到试剂厂家说明书的最低要求时，判断为数据质量合格且可用于结果判读及分析，在判断及分析过程中重点关注微珠荧光信号值等关键指标。

②应保留结果判断分析过程中的所有记录，对于数据质量不合格的样本，分析原因并采取纠正措施。

（一）结果初步分析

1. 判断阴性和阳性质控微珠MFI值符合分析要求，并逐一分析每个样本的结果。当样本的阴性微珠MFI值背景高于实验室可疑阳性阈值；阳性微珠MFI值小于实验室阳性决定值，提示结果不可信时，建议重新采样或样本处理后重新检测。

2. 当分析软件判读结果处于灰区范围，应结合实验室制定的截断值进行人工结果判断，具体分析过程见文献[21]–[22]中相关内容。

（二）结果具体分析

1. HLA等位基因型与抗原型：属于同一个血清学特异性的HLA抗原往往可被数个甚至数十个不同的等位基因编码，如A*02:01、A*02:06、A*02:07等多种等位基因型均可对应A2抗原型。A*02:03对应A203，与A2不同。

2. DSA、非DSA（NDSA）、交叉反应组（cross-reactive group，CREG）、抗原表位及抗原功能表位：若患者血清中检测到抗HLA抗体，依据潜在供者HLA-A、B、C、DR、DQ、DP位点的分型结果，分析得出DSA、NDSA、CREG等结果，并可在报告单上备注。

以接受HLA错配allo-HSCT抗体检测结果为例进行解读：

（1）分析供受者HLA错配位点：患者为HLA-B*35:01，B*46:01，供者为B*40:03，B*46:01，则错配基因型为B*40:03，抗原型为B61。

（2）DSA和NDSA：美国移植与细胞治疗学会（ASTCT）共识建议[24]，使用较敏感Luminex技术SAB，可根据受者与潜在供者存在HLA错配抗原进行DSA分析。若患者血清中检测到B*40:03（B61）抗体阳性，则B*40:03（B61）抗体为DSA，其余多个B位点抗体阳性，均为NDSA。分析得出DSA会根据供者的基因分型而发生变化。

（3）CREG抗体：B7（B*07:02）、B13（B*13:01）、B27（B*27:05）等与DSA-B61（B*40:03）同处于7C CREG的NDSA，均为CREG抗体。

（4）抗原功能表位：抗原抗体结合特异性主要由抗原表位核心区域决定，称为抗原功能表位。通过HLA Matchmaker等工具分析供者含有的非受者自身功能表位个数，可预测移植术后DSA产生的概率，个数越多，产生DSA的概率越大。

（5）对于抗体试剂中未覆盖的等位基因，如未包被B*40:03（抗原型B61）抗原，仅包被B*40:02（抗原型B61）抗原，且供患者错配B*40:03（抗原型B61）等位基因型，建议通过以下方式判断DSA：通过抗HLA抗体分析软件分析B*40:03抗原是否与患者检测到的抗体对应抗原处于核心抗原表位组（Epitope group）。

（二）ASTCT共识推荐方法[24]

1. 使用较敏感Luminex技术SAB，可根据受者与潜在供者存在HLA错配抗原进行DSA分析。

2. 对于接受单倍体供者allo-HSCT的患者，当DSA MFI值>2 000（或单份脐血移植中MFI值> 1 000），在脱敏治疗前后可进行其他方法检测，包括采用天然构象HLA抗原方法，如流式细胞术交叉配型和（或）抗体结合试验，如C1q检测，有助于评估同种异体移植物排斥风险。

三、抗HLA抗体检测结果报告及解读

**专家组推荐：**应根据临床需求对抗HLA抗体检测报告单中的内容进行相应解读，结果报告包括但不限于以下内容：pre-Abs（DSA、NDSA）、dn-Abs（DSA、NDSA）、MFI值、CREG、Epitope group、抗HLA抗体检测结果的变化、抗体治疗效果、动态监测抗HLA抗体时间等。

（一）pre-Abs检测结果的报告和解读

目前国内外文献提示，allo-HSCT前预存DSA的MFI值>10 000，或>8 000，甚至>5 000，是影响allo-HSCT后植入失败和造血重建延缓的高危因素[1]–[2],[25]。故当患者检测到高MFI值pre-Abs，特别是DSA，必要时可在报告单备注告知临床医师关注抗体的再次检测。

（二）dn-Abs检测结果的报告和解读

当患者血清中的抗HLA抗体由allo-HSCT前阴性转变为阳性；抗HLA抗体基因型、抗原型增多，均可解读为产生dn-Abs。当allo-HSCT后产生dn-Abs并持续阳性，特别是DSA或CREG抗体，且MFI值不断升高、抗体类型不断增多时，需重点关注，其与Ⅱ～Ⅳ级aGVHD发生率显著升高，生存率显著降低等相关[8]，应在报告结果时进行提示，并立即与临床沟通，告知抗体检测情况。对于allo-HSCT后检测到抗HLA抗体，但随访中抗体MFI值逐步下降甚至消失，可理解为一过性产生dn-Abs，通过临床随访确定抗HLA抗体是否影响预后。

（三）动态抗HLA抗体结果的报告和解读

1. 应重点关注pre-Abs或dn-Abs高致敏患者抗HLA抗体类型（包括DSA）及MFI值的变化，当抗HLA抗体针对的抗原位点数量及特异性明显减少、MFI值持续下降时，可提示治疗有效。

2. 抗HLA抗体MFI值无显著变化，可能由于患者抗HLA抗体MFI值过高，达到或超过检测上限，抗原抗体结合饱和，无法反映真实抗HLA抗体水平。

3. 患者虽然经抗HLA抗体治疗，但抗体的MFI值无明显变化，建议更换抗体治疗方法或联合多种治疗手段。

4. 部分高MFI值抗HLA抗体下降，但部分原较低MFI值抗HLA抗体有所升高，可能是患者外周血中抗HLA抗体过多，竞争结合抗原性强的微珠。经治疗后原高MFI值抗HLA抗体下降，且MFI值下降趋势较显著时，提示治疗有一定效果，可继续治疗。

5. 采用血浆置换清除抗HLA抗体时，由于仅清除外周血循环池里的抗体，可造成抗体MFI值暂时下降，但随后可能会再次升高；免疫抑制剂的使用等也可造成抗体MFI值暂时下降。

6. 当患者存在上述检测结果时均需要动态监测。当患者抗HLA抗体处于低MFI值且无明显变化时，可适当降低抗HLA抗体检测频率。

（四）抗HLA抗体治疗有效性判断

近年国内外研究结果提示，针对抗HLA抗体治疗有效性的判断均无统一的定论[26]–[29]。苏州大学附属第一医院的一项回顾性研究显示，单倍体allo-HSCT前患者抗HLA抗体（包括DSA）MFI值下降到<5 000或抗体类型减少50％可作为抗HLA抗体治疗较理想的判断依据[28]。抗HLA抗体临床治疗有效性的判读详见《HLA不合造血干细胞移植患者供者特异性抗HLA抗体检测结果临床解读中国专家共识（2025年版）》[30]。

（五）动态监测HLA抗体时间

拟行allo-HSCT患者应动态监测抗HLA抗体。HLA抗体阴性患者可根据移植后的具体情况决定是否监测，建议移植后进行1次抗HLA抗体检测，以判断患者的抗体是否发生变化。无论患者移植前是否有pre-Abs，当患者移植后检测到dn-Abs（包括DSA、NDSA），需要增加HLA抗体检测频率，并关注抗体类型和MFI值的变化。对于allo-HSCT前抗HLA抗体阳性（包括DSA）患者，可参考肾移植指南[31]，在allo-HSCT前，allo-HSCT后第15天、1个月内，各进行一次抗HLA抗体检测。当allo-HSCT患者出现移植物功能延迟、原发或继发性植入不良、造血重建不良、GVHD等临床表现时，在多个时间点（如发生aGVHD后第7、14、28天）动态监测HLA抗体。当患者接受抗HLA抗体（包括DSA）脱敏治疗时，建议在抗体治疗后第1、2、4周的3个或3个以上时间点动态监测HLA抗体及DSA，这对于确定抗体的清除效果至关重要，之后可根据患者情况决定抗HLA抗体监测频率，直至植入和（或）抗体的MFI值明显下降。各移植中心可参照《HLA不合造血干细胞移植患者供者特异性抗HLA抗体检测结果临床解读中国专家共识（2025年版）》[30]相关内容。

应根据中国人群常见HLA等位基因型频率大于0.1％制备HLA抗原谱的抗HLA抗体试剂，避免漏检中国人群HLA常见等位基因和抗原型。未来可对我国高致敏患者进行多中心临床和基础研究，明确影响移植预后的HLA抗体类型和MFI值阈值等风险分层体系，从而选择最优供者、调整预处理方案，对于高致敏患者应告知其移植风险。
